# Development of Technologies for Local Composting of Food Waste from Universities

**DOI:** 10.3390/ijerph17093153

**Published:** 2020-05-01

**Authors:** M. A. Vázquez, R. Plana, C. Pérez, M. Soto

**Affiliations:** 1 Environmental Officer, Department of Environment and Sustainability, A Coruña City Council, 15011 A Coruña, Spain; mv.trillo@coruna.es; 2 Composting Department, Organic Wastes Management Consultancy, 08027 Barcelona, Spain; plana.compost@gmail.com; 3 Revitaliza Plan department, Senior advisor of Revitaliza, Pontevedra Provincial Council, 36071 Pontevedra, Spain; carlos.perez@depo.es; 4 Department of Chemistry, University of A Coruña, 15008 A Coruña, Spain

**Keywords:** decentralized composting, static composters, dynamic composter, food waste, university

## Abstract

The amount of biowaste generated by university canteens (BWUC) in the faculties of the University of A Coruña (UDC) varies between 6 and 100 kg/day. In addition, the gardening services of the campus generate even higher amounts of garden waste (GrW), including pruning, which, once crushed, serves as bulking material for composting the biowaste from the canteens. Decentralized composting has been chosen with the aim of producing high quality organic fertilizers for university urban gardens while reducing the environmental burdens of both waste management and agricultural practice. Small static home composters of 340 L (SHC) for smaller amounts of generation (up to 20 kg BWUC/day) were used, while, for faculties of higher generation (up to 40 kg BWUC/day on average), the first composting stage was carried out in a closed and dynamic composter (DC). The dynamic composter was designed and built specifically for this project and its features were improved and optimized throughout the study. The pilot project was carried out in two centers of the UDC, which are known as the Philology Faculty (PF) and the School of Architecture (SA). All the organic waste generated by the canteens of these two colleges from January 2011 to July 2011 (approximately 3000 kg) was treated. Composting in SHC included a thermophilic phase that extended one month beyond the loading period for which thermophilic temperatures were also recorded. The use of the DC as the first stage in combination with static composters (SC) for the maturation stage reduced the overall thermophilic phase to 6–8 weeks. The complete maturation (*Rottegrade* class IV-V) was achieved after about four months in SHC and after two months when using the combined DC-SC system, if the right conditions of moisture were maintained. The chemical quality of the compost produced was compatible with Class A of Spanish legislation (equivalent to organic farmer quality) and the C/N ratio ranged from 9 to 15 depending on the relation BWUC:GrW.

## 1. Introduction

The production of quality compost from waste and other organic materials treated locally, at the source, has become an important objective for the sustainable and efficient management of urban solid waste and the conservation of soils. Local composting promotes better environmental conditions in the area, the creation of local jobs related to circular economy, and citizen awareness about waste reduction and recycling [1,2,3,4,5,6]. Moreover, organic waste becomes a local resource to produce compost and promote urban agriculture [7]. In addition, incorporating organic matter into the soil favors fertility, stores carbon, limits erosion, favors a better water retention, and facilitates agricultural tasks [8,9,10].

During the last 10 years, the decentralized and local management of biowaste has been applied as a real and complete alternative to the models based on collection and transportation. It is very common in the Basque Country (Spain) where many municipalities developed these models as their only option for biowaste treatment with good social, environmental, and economic results [5,6]. Other relevant territory where local composting is being developed and promoted as the main model for biowaste management is the province of Pontevedra (Spain), where almost 2500 t of biowaste were composted in home and community composters in 2018 in rural and urban areas [11,12]. Another main reference in local composting models that demonstrate they are also suitable in dense populated areas is the city of New York, where, since 2012, it has been developed as an ambitious program to promote local composting through community composting (>200 sites) and several mid-scale composting facilities inside the city, while treating more than 2000 t of organic wastes in 2018 [13].

Currently, local composting is spreading in university campuses, not only as an environmental measure for waste management but also with educational, dissemination, and awareness proposals [14,15,16]. Compostable biowaste can vary widely from 17% to 60% of total campus solid waste. This is due, in part, to the inclusion or exclusion of some waste materials such as paper towels, disposable paper cups, meat, bread, and others from composting [14,17]. Several universities worldwide run their composting programs on their sites since 2000 or even early with universities being more likely to have composting programs than cities in which they are located [17,18]. Early reports refer to composting in windows, aerated bins, or in-vessel experiencing problems of leachate and odor [15,19]. Some of these facilities have been upgraded over the years and most of them are campus-centralized or located outside [14,15,18,20].

The main campus of the University of A Coruña (UDC) is located Elviña-A Zapateira, within the limits of A Coruña city (Galiza, Spain) and conserves areas that still maintain traditional agriculture practices. The UDC set out to contribute to value and preserve this rich heritage and, to this end, the UDC has run a project of urban vegetable gardens since 2013 intended for the cultivation by the members of the University, with both students and staff. The use of these vegetable gardens required high quality organic fertilizers, which must be locally produced in order to guarantee sustainability. The rate of compost addition to maintain soil organic carbon content under Galician conditions was estimated at between 4 and 7 t/ha (dry weight), while, up to 16 t/ha, would be required to reach the desirable 3.5% soil carbon [9]. Needs would be even greater in the near future due to the effects of climate change. However, there is a lack of studies reporting on true decentralized composting programs and technologies on campus buildings, which is on the smaller scale where waste is generated.

On the other hand, different types of organic waste are generated at the UDC campus. These includes pruning, grass cuttings, and other vegetable wastes, as well as kitchen and food waste from various university canteens. All of these organic wastes can be the subject of a joint management and treatment process, in order to produce soil amendment and high quality organic fertilizers. This configures an ideal situation in which two of the goals of the UDC’s sustainability policy are realized: the dissemination and promotion of local-traditional and organic agricultural practices and the sustainable management of organic wastes.

Most of the published research about small-scale composting related to treatment volumes like household composters have been done under operation conditions that are completely different from the normal ones. Composters have been filled at once, watering and turnings have not been done at the adequate frequency, the organic waste is brought from waste treatment facilities to assure it has enough quantities to initiate the experiments, thermophilic temperatures have not been achieved, etc. [21,22,23,24,25,26]. Therefore, at the moment of transfer, these results to predict the efficiency of any local composting model for biowaste management, there are several uncertainties that become crucial to assure the efficiency of the model. Uncertainties that influence process efficiency and the quality of the final product can be related to different control guidelines for specific composting models, on-site management requirements, biowaste type and composition, and feeding rate.

The general objective of this study focused on developing a decentralized composting system for organic waste from university canteen residues placed near each facility. The development of a technology adapted to the needs of the UDC was sought, dependent on the quantities of organic waste generated and other local conditions, which are feasible from the different logistic, economic, and environmental points of view. The investigation has addressed the following particular objectives.

- To quantify and characterize the organic waste of university canteens generated in some centers of the UDC.
- To define an optimum ratio between biowaste generated by university canteens (BWUC) and crushed garden waste fraction (GrW) used as a bulking agent. The proportion experimented initially was 1:1 (BWUC:GrW, in volume), and, later, other relationships were tried.
- To establish the operating routines in two different scenarios: (a) Low generation of BWUC and composting through static home composters (SHC), (b) Medium-high generation of BWUC and application of a dynamic composter (DC) as the first stage.
- To determine the stability of the compost, the time-scale of the process and the different stages to achieve it.
- To determine the quality of the compost.

## 2. Materials and Methods

### 2.1. Inventory of Organic Waste Generated by University Canteens

The University of A Coruña (UDC) involves a population of about 20,000 people, including students and staff. The university activities take place in 20 study centers as well as in another 24 buildings dedicated to research and administration. The UDC comprises eight different campus locations, accounting for a total area of 56 ha. The main campus of the UDC is Elviña-A Zapateira and includes 10 study centers and six research centers in a 41.5 ha area. At the moment of the study, 11 cafeteria-canteen services were located in this campus area.

In the first phase, a survey was carried out in 2009 on those responsible for the university canteens in order to estimate the amounts of organic waste generated in them. The survey was answered by all the services of the Elviña campus (5) and the A Zapateira campus (4). Questions were related to the number of meals served each day of the week by sections to the existence of selective collections and to the volume of organic waste generated. The questionnaire used asked for the amount of generated waste, starting from a minimum of 50 L, and for the qualitative composition of the residues (types of predominant materials, such as vegetables, fish and meat, fruit and oranges, bread, pastries, pastas, and rice leftovers, and coffee grounds.

In addition, during the entire period of the composting study (about four months in 2011) for two of these university canteens, a daily inspection of the residues was carried out in terms of the quality of the separate collection and determination of the weight and volume of the quantities generated. The obtained data allowed us to prove the validity of the questionnaire used.

### 2.2. Composting Units and Operation at the Philology Faculty

In the Philology Faculty (PF), three SHC of 340 L (Container KOMP 340 model) were used. Every day, the BWUC and the bulking agent were added to one of the composters and this material is mixed manually in the same composter. This mixing only affects the newly added organic materials and that of the two last additions, which limits the action only to the top of the composter. The loading process of one of these composters extends for a period of between 1 and 2 months, and, during this period, one or more full turns to mix all the material was carried out. The composter is fed until it is full. When this happens, the operation of a second composter that will receive fresh waste begins, and the process was followed as in the first composter. After an initial composting period in the first composter, when the temperature dropped at least to the mesophilic range, the material was transferred to a third composter for the maturation stage. In this way, an additional and complete mixture of all the material from the first composter and the correction of the moisture were realized and the process continued until total stabilization. The first composter was empty to receive fresh waste once the second composter was full. If necessary, the moisture content was corrected throughout the process by manual watering.

### 2.3. Composting Units and Operation at the School of Architecture

**Figure 1:**
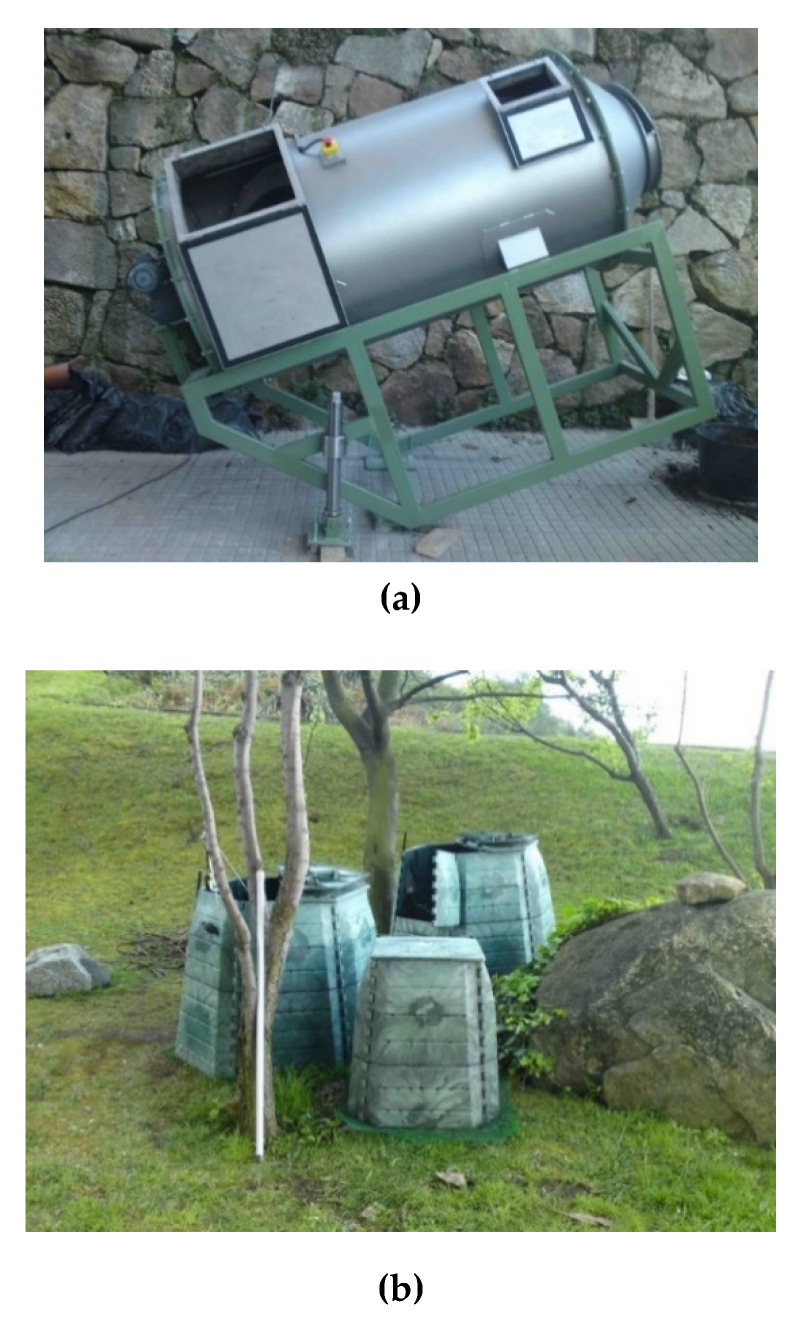
Composting system at the School of Architecture: (**a**) First step Dynamic composter, Plana^®^ design, (**b**) Maturation static composters (2 Container KOMP 1050 units and 1 Container KOMP 340 unit).

In the School of Architecture (SA), a DC was used as the first stage of the process (Figure 1). The dynamic composter is formed by a cylinder of 1 m of diameter and 2 m of length, with the following characteristics.

- A capacity of approximately 1.5 m^3^ and 40–80 kg/day.
- Automatic and programmable system of mixing and advance of the material by the action of an endless spiral (turn over) powered by an electric motor.
- Loading entrance and unloading exit.
- Pre-installation of sampling points for measuring the process conditions (T, pH, O_2_, moisture content, etc.)
- Forced aeration system with the biofilter to treat the gaseous effluent.
- Leachate collection system.
- Manual adjustment system of the inclination of the main body (the cylinder, which allows control of the level, and, consequently, the speed of advance of the waste).

The DC was loaded daily or every two days with fresh waste along with the bulking agent material. Mixing and aeration mechanisms were regulated with electronic timers. A mixing frequency of every 1–2 h was applied in the DC, which provided an intense axial mixture and the longitudinal advance of the material following a piston-flow behavior. The estimated retention time of waste within the digester was about 10–20 days, as estimated from punctual measurements.

The pre-digested material removed from the dynamic digester goes to a static composter (SC) of 1050 L (Container KOMP 1050 model), or to a plastic mesh bag of 800 L (big-bag) where thermophilic temperatures are still reached for several days, to continue with the maturation stage at decreasing temperatures. This unit was named as a static maturation composter (SMC), which refers to both the KOMP 1050 and the Big-Bag. If necessary, the moisture content in SMC units was corrected throughout the process by manual watering.

In the SA, the process was monitored for approximately four months. In the first month, the operation of the dynamic composter was evaluated in its initial configuration (DC1). In configuration DC1, the reduced power of the mixing and advancing system limited the use of the digester volume to below 50% of its capacity. After this period, the power of the engine was increased and the new Configuration 2 (DC2) was used continually for three months in order to obtain steady state operational parameters.

### 2.4. Comparison of Dynamic and Static Composting Processes

For a better and greater understanding of the processes in the dynamic composter, the operation of the dynamic composter in configuration DC1 was compared with the initial operation of a static composter, both working in parallel under similar conditions of loading rates and waste composition. To this end, the Container KOMP 1050 model, a common static composter of 1050 L (SC1050), was used. Throughout the first 29 days of the experiment, the same fresh material (fresh BWUC from the SA mixed with GrW at the ratio of BWUC:GrW 1:1 in volume) was provided to the DC1 and the SC1050. The amounts fed during this period were 285 kg to the dynamic digester, and 283 kg to the static composter in both cases with a density of 0.46 kg/L. In this way, the total volumes of the mixture added were 617 and 614 L to the DC1 and SC1050, respectively. The operation of the DC1 was carried out as described in Section 2.3. On the other hand, the complete mixture in the SC1050 took place two times per week during the rest of the time at rest.

In addition, samples from the DC2 working at the steady state were compared with samples from the SMC composter that received the material from the DC, after a long maturation phase carried out through vermicomposting.

This comparison aimed to shed light on the process characteristics in the DC1 and to determine the distance to a more stable and mature compost. The physical properties of material in process in both the dynamic and the static composters were determined, including basic parameters such as the content in volatile solids (VS) and moisture content (MC). Other parameters were obtained by the suction table method described below (Section 2.5). These were: dry bulk density (BDdry), wet bulk density (BDwet), contraction value (Cv), and water holding capacity (WHC).

Lastly, several derived parameters, that is, particle density (PD), total porosity, or void space (ϕ), air capacity (AC), and free air space (FAS), were obtained through the following equations [27,28].

PD = 1/[(VS/100) / 1550 + (1 − VS/100) /2650](1)

ϕ = (1 − BDdry/PD) × 100(2)

AC = ϕ − WHC(3)

FAS = ϕ (1 − MC/100)(4)

where all the parameters are expressed in percentage (%), except PD and BDdry (kg/L).

### 2.5. Sampling and Analysis

The temperature of the material in composting was routinely determined at three points of each mass to obtain the average value. Oxygen in the interstitial atmosphere was determined in two points both in the DC and in the static composters SMC and SC in the SA. Moisture was routinely qualitatively checked, and MC was occasionally tested in the laboratory. On the other hand, samples of both the compost and the raw materials (bulking agent and BWUC) were collected for the determination of different parameters. Analyses of the final product were also performed to determine their quality (stability, heavy metals, percentage of C and N, nutrients, presence of improper materials, etc.). The volume of leachate generated in the DC was also determined. Stability in the intermediate stages of the process and the final compost samples was determined by the *Rottegrade* test (Dewar Self-Heating Test) and by respirometry [29].

MC was determined by drying to a constant weight (24–48 h) in an oven at 90 °C and volatile solids (VS) by ignition at 550 °C (4 h). The analysis of the nitrogen content (N), total carbon (C), and total organic carbon (TOC) were performed on an EA1108 elemental analyzer (Carlo Erba Instruments, Milan, Italy) equipped with an AS200 autosampler. For nitrogen and carbon analysis, samples were weighed in tin capsules. For organic carbon analysis, samples were weighed in silver capsules and carbonate was removed by addition of HCl before the analysis. Samples were oxidized by combustion at 1020 °C using an oxidation catalyst containing Cr_2_O_3_ and AgCO_3_O_4_. After combustion, the resulting gases passed through a reduction reactor containing elemental copper at 650 °C. After removal of water, the evolved N_2_ and CO_2_ were separated by gas chromatography and detected by thermal conductivity. All samples were analyzed in duplicate. The quantitative analysis of metals was performed in triplicate in samples of 0.5 g (from a sample of approximately 100 g, previously dried, and ground to homogeneity) by adding 10 mL of distilled cc HNO_3_ and heating to 175 °C for 10 min, according to the US EPA 3051A method [30]. All metal concentrations were determined using inductively coupled plasma mass spectrometry (ICP-MS Element XR or Element2 from Thermo Electron, Bremen, Germany).

Physical properties of the material in process were determined using a suction table with a sand bed [31], where pressures of −50 cm and −10 cm were applied to the pre-conditioned and saturated samples before proceeding to dry them in an oven.

In order to facilitate interpretation, relative heavy metal (HM) values are defined as the ratio of the metal concentration in the compost sample to the Class A regulation limit [32].

Relative HM concentration = C_i_/C_Ai_ (dimensionless value) (5)

where C_i_ is the concentration of metal i in sample compost and C_Ai_ is the concentration limit of metal i for Class A in the Spanish regulation (RD 865/2010 and RD 506/2013).

Unless otherwise stated, mean and standard deviation values were used to assess the characteristics of compost samples. The suitability of the least-squares fit (linear regression) was evaluated by the square of the coefficient of determination (R^2^), the statistical *F*-value, and the probability (*p*). Statistical analyses were carried out in Microsoft Excel (Excel 2010 v. 15.0.4875.1000, Microsoft Corp., Redmond, WA, USA).

## 3. Results and Discussion

### 3.1. Quantities and Characteristics of the Waste Generated

The UDC has 17 cafeteria-canteen services of which 11 are located on the Elviña-A Zapateira campus, in a radius of less than 1 km, and they cover approximately 80% of the total university service. The estimation study through surveys provided the information of nine of these canteens in Elviña-A Zapateira shown in Table 1. This data indicates that the generation of organic waste by the university canteens (BWUC) can vary between 6 and 50 kg/day on average for each one of the centers, even though peaks of 100 kg of BWUC could be produced in a single center a day. In this way, 1275 kg of organic waste were estimated to be generated every week in Elviña-A Zapateira in 2009.

The weighing campaign carried out in the PF and the SA during the development of the present study (2011) showed the following results (considering school days, that is, five days a week):

- PF: generation of 8.0 ± 3.6 kg/day
- SA: generation of 37.1 ± 7.4 kg/day

These results show a 32% higher generation in SA and a 23% higher generation in the case of PF, in relation to the estimate made two years earlier. Regarding the type of organic material that constitutes BWUC residues, food leftovers were predominant (44–91% of total BWUC for four centers) while waste from food preparation, squeezed oranges, and coffee grounds were usually present but in variable amounts depending on the center (0–37%). In this way, the processed food always remains as the majority fraction, while other types of materials show a very variable presence. The presence of inappropriate materials such as plastic and metal, mainly bottle stoppers, plastic wrap, sauce, and condiment bags or kitchenware) was measured and was very rare or sporadic. Physical contaminant materials was always far below 1% of raw material.

The chemical composition of the waste materials used for composting is presented in Table 2, including the BWUC from SF that was not included in the composting program at that time. The content of certain nutrients was variable in BWUC and more reduced in the case of the bulking agent. The bulking material had a relatively high C/N ratio (value of 53), which is due to the presence of branches and leaves between crushed plant remains, along with more woody trunks. Composting of the organic waste generated by university canteens, with C/N ratios of 14–21 (BWUC from SA, PF and SF, Table 2), need a carbon-rich amendment to balance the C/N ratio and, above all, a structural material that facilitates the aeration of the matrix during the process. The used bulking material provided both the C/N ratio and the structure correction. Lastly, Table 2 also indicates the content of heavy metals in the initial materials. With the exception of Cd in organic waste from the Science Faculty (SF) canteen, the metal content in all the samples is very low, which allowed us to expect a compost of good chemical quality.

### 3.2. Composting in the Philology Faculty

**Figure 2:**
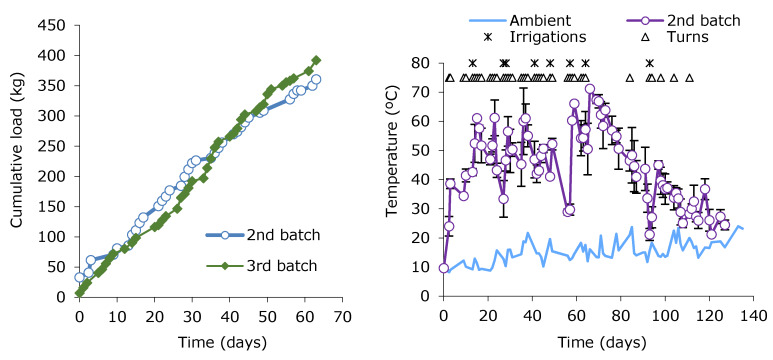
Cumulative loads (BWUC + GrW) on the left and temperature evolution on the right in the Philology Faculty composters. Data corresponds to the second and third batches (loads) and second batch (temperature).

The experimental study in the Faculty of Philology lasted for eight months. During this period, three successive composting batches were carried out, in accordance with the process described in Section 2. Figure 2 shows the evolution of the second and third batch load as well as the evolution of the temperature, together with the frequency of turns and irrigations during the second batch. After 63 days of loading, the composter of the second batch kept the thermophilic temperature for over an extra month, which, subsequently, dropped from day 120 to values close to those of ambient temperature. The first and third batches showed similar behavior with only minor variations (data not shown).

Table 3 summarizes composting parameters obtained from the second batch in the Philology Faculty. The loading of the second batch in the PF extended for 63 days in which a total of 35 load episodes took place. Including BWUC and bulking agent, a total of 360.5 kg were added, in ratios of 8.5:1 and 1:1 BWUC:GrW in mass and volume, respectively. An average loading rate of 7.7 kg/day was obtained. After the completion of the 63-day loading period, the material continued to be composted for another 60 days. In the overall period, the temperature was above 65 °C for four days, and higher than 55 °C for 18 days. It means that it fulfils the requirement associated with compliance and the regulations of Animal By-Products (ABPs) in the international legislation in terms of community composting. The process also meets the requirements of the regional rules for sewage sludge composting [33]. The mean thermophilic temperature for the period between the days 11 to 92 was 51.5 ± 9.5 °C (mean ambient temperature of 14.6 ± 3.6 °C). Regarding stability, self-heating assays at day 84 of operation indicated Class II, while a very stable Class V material was obtained on day 129.

**Figure 3:**
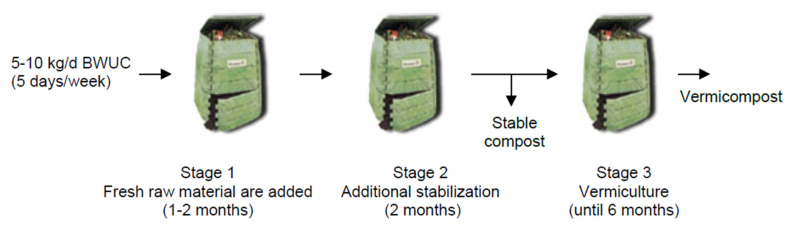
Composting process diagram at the Philology Faculty (Composter volume: 340 L).

According to these results, a three-stage process was proposed as indicated in Figure 3. The complete stabilization of the compost was verified after stage 2 (Table 3). The process, including the periods of progressive loading and thermophilic composting, requires three to four months to produce stable compost (stages 1 and 2). In this way, stage 3 constitutes an optional stage, in the case of wanting to make vermicompost with the aim of increasing the degree of humification of the material. It may also be considered as an additional maturation stage of interest in the case of sufficient space and if the immediate use of the compost is not necessary.

### 3.3. Composting in the School of Architecture

#### 3.3.1. Dynamic Composter at Low Loading Rate (Configuration DC1)

Upon receipt of the DC, provided by Plana Compost^®^, several preliminary tests were performed to demonstrate the performance of all operating mechanisms, including mixing and ventilation functions. Ventilation was found to be necessary not only for oxygenation, but mainly to remove excess water vapor that otherwise condensed and generated leachate and excess moisture from composting material. In addition, it was considered necessary to shorten the width of the spiral blades to avoid the excessive mixing effect and excess effort required to move the endless spiral of the digester. The DC was then continuously operated for one month (DC1 Configuration, described in Section 3.3.2). The engine torque was then modified and continuous operation was continued (DC2 Configuration, Section 3.3.3).

**Figure 4:**
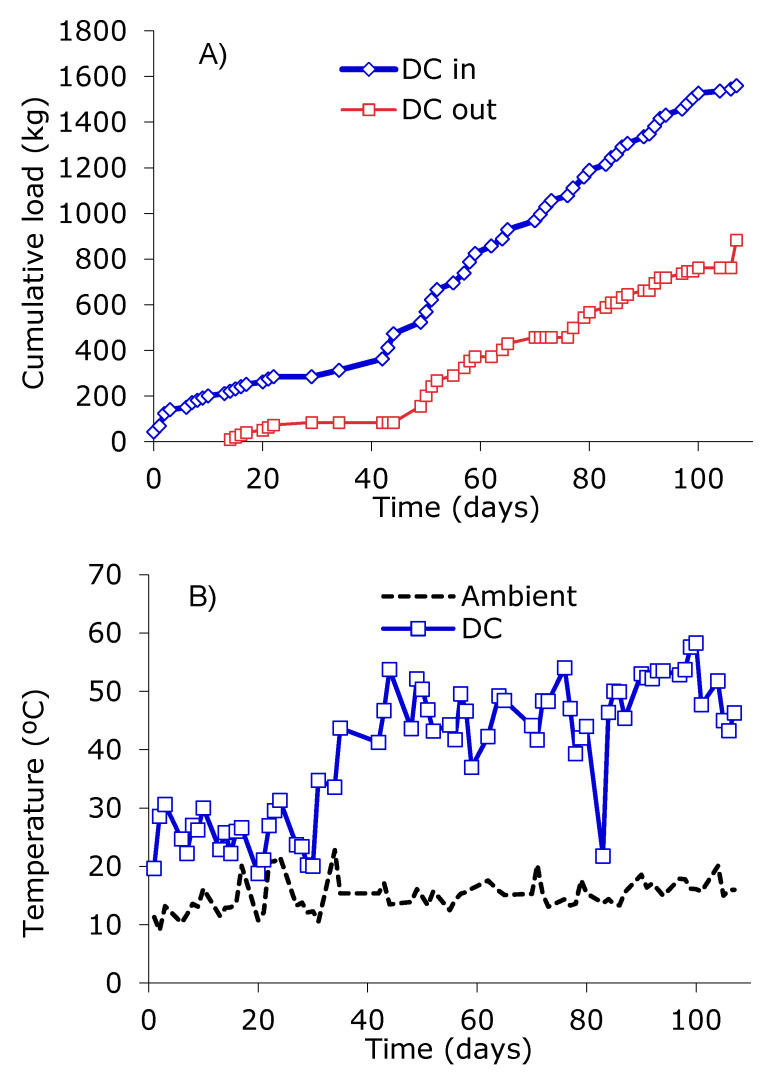

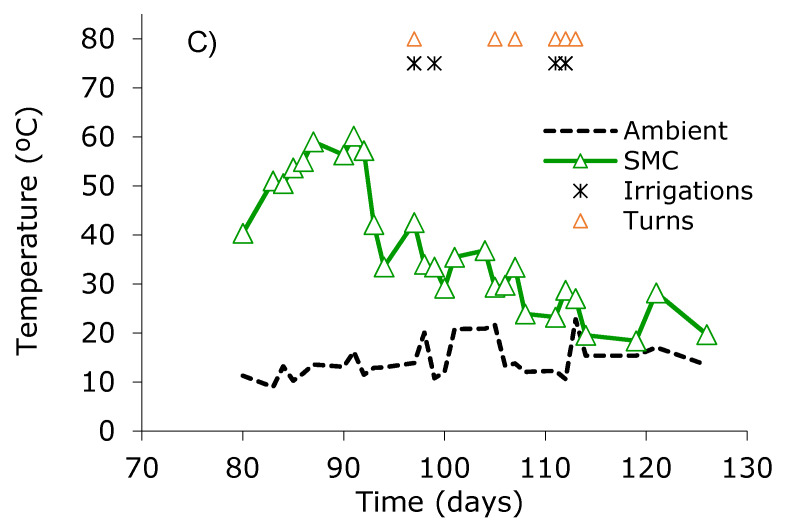
Operation of the dynamic composter (DC) and the static maturity composter (SMC) in the School of Architecture: (**A**) Evolution of the load and discharge applied to the DC, (**B**) Temperature evolution in the DC, (**C**) Temperature evolution in the SMC. For SMC, only one unit is shown, which is the one that received the output material from DC between days 78 and 92 of its operation (Figures A,B). The behavior of other batches was similar.

Figure 4 presents the loading rate to the dynamic composter and the evolution of the temperatures in both the dynamic and the static maturation composters. The first experiment of continuous operation of the DC1 (dynamic composter in Configuration 1) allowed us to check different aspects of the operation of the aerobic digester at a low load (days 0-30, Figure 4A). In the first days, the digester was loaded at a higher rate, reaching about 200 kg of material in total. Above this weight, it was found that the mixing system stopped working due to a lack of sufficient power to move and drag the material by the endless spiral. This forced the removal of material (Figure 4A) and ensured a limit of 200 kg. The loading rate of 13.5 kg/day on average during the period had to be reduced to no more than 10 kg/day. In these conditions, the operation took place at a temperature in the range of 25–30 °C (Figure 4B), even though there was an advanced fermentation process and a significant reduction in volatile solids (data not shown). It was generated as leachate at a rate of 1–2 L/day, showing low pH of 6.3 and high electrical conductivity and ammonia content, of 6.3, 12.4 mS/cm, and 442 mg N/L on average, respectively.

In contrast to the temperatures in the range of 25–30 °C for the DC1 (Figure 4B). The SC (parallel test) reached thermophilic temperatures between 55 and 65 °C from the second day of the operation. This indicates that the low temperatures obtained in the DC1 were consequential for the operation of this unit and not of the characteristics of the waste fed. Likewise, it was found that, in these conditions, once the outlet material of the DC1 has been transferred to a static maturation composter, the material reached and maintained thermophilic temperatures for a period of approximately 15 days.

The low temperatures in this initial phase of the DC1 could be due, in part, to the low load allowed by the mixing system. With a maximum of 200 kg inside, occupying approximately 400 L, the DC1 had unused three-fourths of its volume, which increases the heat losses. On the other hand, for these conditions (maximum load of 200 kg), a maximum retention time of 14 days was obtained and the system could not receive more than 10 kg/day of organic waste.

#### 3.3.2. Static Composter SC1050 in SA

The SC1050 was operated in parallel with the DC1 described in Section 3.3.1. The planning of the experiment was detailed in Section 2.4. The planed 1:1 volume ratio of BWUC:GrW resulted in a mass ratio of 3.1. An average loading rate of 13.5 kg/day was obtained, which is the same as for DC1 but higher than that applied in SHC in the PF. Differences in behavior regarding the SC of PF were scarce. After the completion of the 21-day loading period, the material continued to be composted at thermophilic temperature until day 56. During the period of 1 to 56, the mean temperature was 48.0 ± 11.8 °C (mean ambient temperature of 14.4 ± 3.3 °C). In the same period, the temperature was above 60 °C for at least eight days. The temperature progressively decreased staying below 25 °C after three months of the process.

#### 3.3.3. Dynamic Composter at a Medium Loading Rate (Configuration DC2)

Correcting this initial design of the DC1 would require greater capacity and power to its mixer system. Modifications carried out affected the mixing mechanism and the torque of the engine. These changes allowed to reach about 400–500 kg inside the digester. These conditions were applied from the fortieth day of operation forward (Figure 4A,B), reaching an average feeding rate of about 20 kg/day. In these conditions, the material inside the digester reached about 800-900 L of volume, which was a reasonable use of the total reactor volume (about 50–60% of it). The generation of leachate was completely avoided after the first week of this period.

Under the new configuration during this second period (days 42 to 107), a total of 1291 kg (2809 L) of BWUC and GrW were fed into the DC2 at a rate of 19.6 kg/day on average. The DC2 achieved a mass reduction for the material in the process of about 35.7%, which generated an outlet rate of 12.6 kg/day that was transferred to the SMC. In this condition, the DC2 reached temperatures in the range of 41–54 °C (47.4 ± 6.3 °C on average), which favored the composting process. The material taken out from the digester, in terms of stability, shows a variable *Rottegrade* class II-IV.

The material taken out from the two reached thermophilic temperatures of 52–59 °C in the SMC for 2–3 weeks (the batch in Figure 4C registered 55.4 ± 3.5 °C on average for 10 days), to approach the ambient temperature after four weeks. Various mixing actions and correction of moisture content were done for at least three weeks without a noticeable increase in temperature (Figure 4C, day 108 onwards). Stable compost of *Rottegrade* class IV-V was obtained after 3–4 weeks in the SMC when the DC2 is used as the first stage.

**Figure 5:**
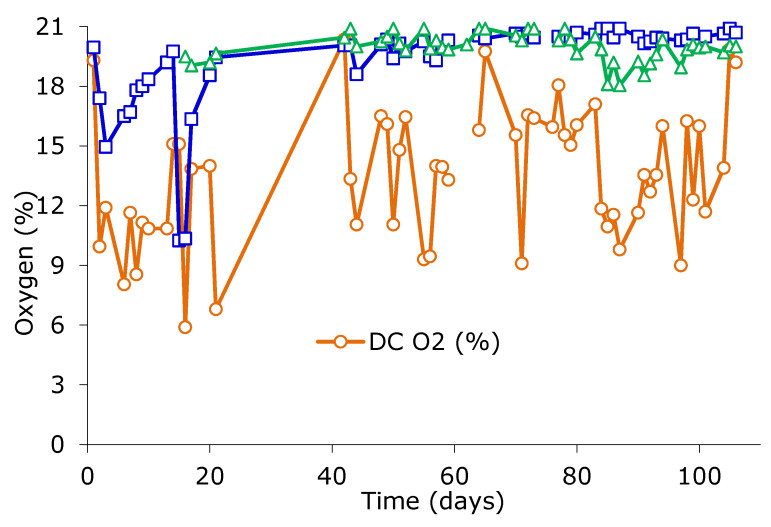
Oxygen concentration in dynamic (DC) and static composters (SC receiving fresh BWUC, several SMC batches receiving the material from the DC).

In general, a good oxygenation of the material in composting was observed, both in the DC2 as especially in the SC and SMC (Figure 5). The lowest oxygen values were recorded on a regular basis in the DC2, especially at the low load stage, which showed values in the range of 8% to 17% oxygen, with an average of 11.4 ± 3.3% (*n* = 15) in the period of low load (days 1–21) and 14.1 ± 3.2% (*n* = 42) in the period of high load. Static composters showed 19.4 ± 2.2% oxygen on average (*n* = 56, SC) and 20.0 ± 0.7% (*n* = 46, SMC).

#### 3.3.4. Steady State Operation of DC2 and SMC at the School of Architecture

Subsequently, all the BWUC of the SA was fed into the DC2, which allowed checking its operation with loads of up to 40 kg/day. The mean loading rate in the autumn 2011 (October to December) was 28 kg BWUC/day, showing similar operating characteristics and results. Thus, the treatment capacity of the DC2 was in the range of 13–27 kg BWUC/m^3^·day (18.5 kg BWUC/m^3^·day on average). With these high loads, during the autumn, the discharge of the digester was made directly to a big bag in which the maturation continues for a period of 3–5 weeks, and becomes, after this period, stable compost. The use of the big-bag facilitates the draw out of compost and transportation to the area of use as well as traceability of the batches.

Earthworms were applied to some SMC batches after the end of the thermophilic phase, which required a period of several months and periodic supervision to maintain high moisture with the need for watering during the summer months.

**Figure 6:**
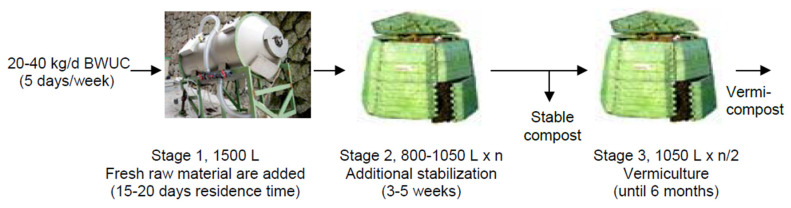
Composting process diagram at the School of Architecture. The static maturity composter (SMC) in stage 2 could be replaced with a breathable bag (big-bag). The number of units in parallel in stage 2 ranged between 2 and 4.

Figure 6 summarizes the results obtained in the SA in terms of 3-stages proposed and their duration. As for the PF system based on static composters, the complete stabilization of the compost was verified after stage 2. The process requires 5–8 weeks to produce stable compost (usually class IV). Thus, the time the material must be in process has been reduced to about half the 3–4 months required by the use of static composters, which we can see by comparing Figure 3; Figure 6.

### 3.4. Physical Properties of Material Samples from Dynamic and Static Composting

The physical characteristics of different samples from the dynamic and static composting units are shown in Table 4. In the initial degradation experiment carried out with the initial configuration of the DC1 and the SC1050, VS content decreased in both units in time because of the effect of degradation. MC was high in the DC1 without watering while it was very low in the SC1050 on day 14 and higher on day 29 due to watering. On the other hand, samples from the steady state operation showed higher VS content and lower MC in the DC2 in comparison to DC1 because of the improved operation of the dynamic composter in configuration 2 and the application of a higher loading rate. The sample from the maturing stage, SMC, had a high MC because of watering to favor the growth of earthworms.

Regarding the evaluation of physical properties of compost samples (Table 4), the values of VS and MC should be considered as independent parameters while some of the parameters in Table 4 are mathematically related, as indicated by Equations (1) to (5) (Section 2.4). A first look at Table 4 as well as the linear correlation graphs indicated that the DC1 sample from day 29 frequently appeared as an anomalous value. DC1 on day 29 was considered to be in a steady state for the conditions in Configuration 1, as 29 days was approximately twice the estimated retention time. On the contrary, DC1 on day 14 was still in an early evolutionary situation.

Thus, the correlation between variables was performed with all data and excludes the DC1_29_ sample in order to better verify the differences. The results are shown in Table 5. The number of significant correlations (*p* < 0.05) was 12 without the DC1_29_ sample and 11 including it. The main differences were found for the parameters BD_dry_, BD_wet_, ϕ, and AC. Significant correlations of DB_dry_ with FAS or with MC without DC1_29_ sample as well as for ϕ with PD and with VS was lost when this sample was included. The opposite occurred for the correlation of ϕ with DB_dry_, DB_wet_, and AC.

Cases of nonlinear correlation were obtained for some parameters. In fact, BDwet and WHC increased and AC decreased in a linear manner with MC up to 65% and then continued to vary while MC asymptotically approached the limit value of 70%. Thus, other factors than MC determined the behavior of these parameters. The same behavior was observed for these variables against FAS for which the asymptote was the lower value of 28% FAS. This was due to the strict linear relation between FAS and MC in the operating conditions.

In the initial configuration DC1, the material had the largest bulk densities, BD_dry_ and BD_wet_, and WHC, while showing low AC and FAS values. Among these parameters, only low FAS was explained by the high MC, while the others were on the asymptote for MC (i.e., the zone near 70% MC or water saturation zone in which these parameters change without following MC). Configuration DC2 improved these properties that approached those obtained for static composters (SC1050 and SMC). DC1 samples showed a greater compaction of the material, which was indicated by lower contraction capacity, and lower AC values, giving the material a mushy appearance that disappeared in configuration 2 (DC2). In fact, with the change in configuration, the AC increased from values below 35 to values above this threshold, which was indicated as convenient for composting [34] and similar to that of static composters (Table 4).

All the samples analyzed had high or very high ϕ values (Table 4). Compost samples can be classified as high or having very high porosity, depending on whether ϕ is higher than 80% or 90%, respectively [34]. ϕ values obtained for the samples SC and SMC are higher than those of the DC1 and DC2 samples. In general, the ϕ increased throughout the composting process, as the VS decreased. However, for DC1 between 14 and 29 days, ϕ decreased rather than increased. In this period, the process of biological degradation that decreased VS (from 80.4% to 77.2%) did not increase porosity, perhaps due to a faster and more intense physical phenomenon that led to increased dry density and reduced particle size [35]. The data suggests that mechanical mixing in the DC accelerated substrate hydrolysis, reduced the particle size, and shortened the composting time. This occurred in both DC1 and DC2 configurations. If a medium or high loading rate was applied, which required configuration DC2, the heat generated counterbalanced the energy losses and the DC reached thermophilic temperatures. However, if low loading rate or low mass content in the digester occurred (configuration DC1), the temperature would remain low, in the mesophilic range.

Cv does not correlate with any of the other variables (R^2^ < 0.11), and there is no difference between DC and SC samples, even though the values increase with composting time in both cases. It was in the 5% to 14% range, so it may be considered low but appropriate.

The analyzed composts show high or very high values of AC, except for the DC1_29_ sample. None of the samples presents risk of asphyxiation, in concordance with oxygen profiles (Figure 5). For the DC2 conditions, AC and WHC parameters appeared equilibrated and similar to that of values for SC samples.

FAS correlated strictly with MC (R^2^ = 0.997). The values of FAS obtained for the SC samples were above the optimal range (30–35%) proposed for composting [28,36]. This was due to the low MC caused by the high temperatures and intense evaporation that would require more intensive watering practices. Given the high oxygen concentration in SC material during the thermophilic phase as well as the high AC values, all these data suggest that a finer bulking material could be used. A finer bulking material will reduce the natural ventilation, which, in turn, would reduce water evaporation. However, there would be an increased risk of anaerobic conditions, which is a potential but absent problem under the conditions applied.

For the case of the samples DC1, the values of FAS are slightly below the optimum range with the values obtained around 28%. This fact, associated with a high MC and low AC, could lead to anaerobic conditions that slow down the composting process [37]. However, oxygen content (Figure 5) indicated no oxygen limitation and it indicated that the bulking agent material used was appropriate for good oxygen diffusion in the operating conditions.

### 3.5. Compost Quality and Overall Evaluation

Table 6 shows the characteristics of the final samples from two SA and PF compost batches. The compost has a high fertilizer power with 2.5–3.6% nitrogen content and C/N ratios between 11–15. The lowest C/N ratio and higher content in N of the PF sample is partly explained because a lower proportion of the bulking agent material was used. These C/N ratios, particularly, PF compost indicate a good compost maturity as well as a good N conservation [38,39]. The nutrient content was in the range of values previously reported for domestic composting programs [40]. Phosphorus content is similar or somewhat higher than that found in other industrial composts and in home composts. The values of K and Mg in Table 6 were lower than those reported by Vázquez and Soto [40] for home composts while the values for N and Ca were higher.

**Figure 7:**
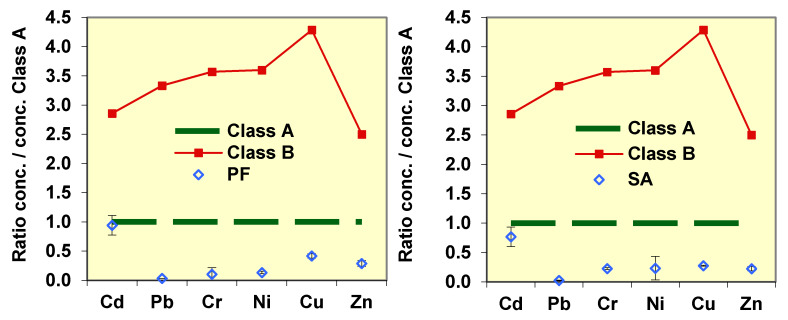
Mean concentration (± S.D.) of heavy metals in the final compost from PF and SA. Concentrations are expressed as values relative to the legal and stricter Spanish limits for Compost Class A, and its comparison with the limits for Class A (relative value = 1) and Class B.

In relation with the chemical quality, heavy metal concentrations are generally low, although Cd is close to the limit value of class A (Figure 7). Cadmium is present in food waste, so a process with a low proportion of bulking agent material or with a very advanced degradation of organic matter can lead to values that exceed the limit of class A. In addition, there has been no *Salmonella* and there were 321 cfu/g of fecal coliforms, which would be compatible with class A of the US legislation on biosolids EPA 40 CFR Part 503 [41].

The high quality of the obtained compost indicated that the separation of organic waste at the source in these university canteens was implemented with very good results. The presence of inappropriate materials (or physical contaminant materials such as plastic and metal) was very rare or sporadic, which was always far below 1% of raw material. The obtained results also emphasize the satisfactory participation of the different agents involved, from the staff of the cafeteria services, to the gardening company. The total of the organic waste generated in these two centers, just over 5000 kg in 2011, together with approximately another 2000 kg of crushed plant remains, has been transformed into compost. The obtained compost was used as fertilizer in the university vegetable gardens cultivated by students and staff.

Once the system had been implemented, the personnel in charge of the supervision of the equipment and composting areas dedicated 1 h a week to each composting area. This included students with environmental scholarship, combined later with job placement workers provided by a charitable non-governmental organization. The composting process in static composters has been evaluated as simpler for reduced amounts of organic waste generated by university canteens, up to 15–20 kg/day. In this range of operations, in the following years, new composting areas were installed in which static composters of different volumes were used, which varied from 340 to 1400 L. At the end of 2019, nine composting areas were in operation on the UDC campuses, which treated approximately 80% of the BWUC generated [42]. For higher generation rates, it was considered convenient to use a mechanical dynamic composter that performs the work of mixing and turning of the material during the first high rate composting phase. The dynamic composter available in the SA facilitated and accelerated the process.

As suggested by Valentukevičienė et al. [43], these on-campus composting systems are also a valuable opportunity for student participation in research and internship activities through teamwork with projects in various study subjects. In practice, they have provided research practices for undergraduate and doctoral students [44].

## 4. Conclusions

The operation of the static composters was very simple and reliable when the bulking agent was of a suitable and homogeneous particle size as used in these experiments. The system is feasible for the treatment of amounts up to 20 kg/day of organic food waste dedicating 1 h of labor per week. For higher amounts of waste, the use of the dynamic composter that favors the mechanical and automatic mixing of the waste and the bulking materials was considered desirable. This dynamic composter also accelerated the thermophilic fermentation stage.

In this study, the limitations of the dynamic composter in its initial configuration were identified and several improvements have been proposed and applied to achieve thermophilic temperatures. In the initial configuration, low temperatures and high moisture content appeared to cause higher bulk densities and very low free air space. These features were compatible with an acceleration of the process caused by a rapid hydrolysis and reduction of the particle size in the dynamic composter. When the load capacity of the dynamic composter was increased, these parameters improved and approached those of the static composters but maintained the effect of the dynamic composter on the process acceleration. The treatment capacity of the dynamic composter stood at the range of 13–27 kg BWUC/m^3^·day.

The study established the routines and minimum times of operation depending on the different systems used. Stable compost of *Rottegrade* class IV-V was obtained after 5-8 weeks when the dynamic digester is used as a first stage, and, after 3–4 months, when only static home composters are used. The final compost demonstrated a high-fertilizing power with nitrogen content of 2.5–3.6% and C/N ratio of 11–15 depending on the proportion of the bulking material to waste used. The concentrations of heavy metals were low, so the compost can be classified as a Class A, according to current regulations. Cadmium, which is present in food waste, was located near the limit value of class A so that a process with a low proportion of bulking material combined with a very advanced reduction of organic matter could lead to values exceeding the limits of class A.

## Figures and Tables

**Table 1:** Waste generation produced in the canteens of the UDC campus at Elviña and A Zapateira.

	A Zapateira Centers ^a^				Elviña Centers ^a^					
Concept (Amount kg/week)	SA	STA	SF	PF	SEF	EBF	TSE	CSF	CC	TOTAL
Weekly Menus (nº)	465	595	330	140	585	1010	300	275	920	4620
Fresh Raw Material	263.8	175.0	125.0	19.1	221.6	152.8	170.2	187.2	348.4	1663
Fresh Peeled Raw Material	0.0	150.0	94.7	47.6	167.9	611.1	56.7	0.0	264.1	1392
Pre-Cooked Raw Material	87.9	125.0	29.8	38.1	52.7	0.0	0.0	20.8	82.9	437
Total Raw Material	351.7	450.0	249.6	105.9	442.4	763.9	226.9	208.0	695.8	3494
Waste Fresh Raw Material	118.7	78.8	56.2	8.6	99.7	68.7	76.6	84.2	156.8	748
Waste Fresh Peeled Raw Material	0.0	45.0	28.4	14.3	50.4	183.3	17.0	0.0	79.2	418
Waste Pre-Cooked Raw Material	22.0	31.3	7.4	9.5	13.2	0.0	0.0	5.2	20.7	109
Total Organic Waste	140.7	155.0	92.1	32.4	163.3	252.1	93.6	89.4	256.8	1275

^a^ SA: School of Architecture. STA: School of Technical Architecture. SF: Science Faculty. PF: Philology Faculty. SEF: Science Education Faculty. EBF: Economy and Business Faculty. TSE: Technical School of Engineering. CSF: Computer Science Faculty. CC: Central Canteen.

**Table 2:** Nutrient elements and heavy metals present in the samples of garden waste used as a bulking agent and organic waste from different UDC centers.

Material ^a^	GrW	BWUC SA	BWUC PF	BWUC SF
**Mg (g/kg)**	1.07	1.22	0.848	1.13
**P (g/kg)**	1.76	3.33	5.98	3.03
**Ca (g/kg)**	10	45.8	13.4	14.8
**K (g/kg)**	4.44	11.5	10.1	13.16
**N (%)**	0.85	2.63	3.18	1.96
**C (%)**	44.69	42.48	49.74	43.80
**TOC (%)**	42.55	38.74	45.13	40.42
**C/N**	52.6	14.7	14.2	20.6
**Cd ^b^**	<0.1	<0.1	<0.1	0.151
**Hg ^b^**	<0.03	<0.03	<0.03	<0.03
**Pb ^b^**	1.29	<0.78	<0.78	<0.78
**Cr ^b^**	<0.78	<0.78	<0.78	<0.78
**Co ^b^**	<0.3	<0.3	<0.3	<0.3
**Ni ^b^**	<0.78	<0.78	<0.78	<0.78
**Cu ^b^**	6.9	3.44	2.82	5.6
**Zn ^b^**	23.9	16.5	19.7	15.1
**As ^b^**	<0.3	<0.3	<0.3	<0.3
**Se**	<0.3	<0.3	<0.3	<0.3

^a^ GrW: Garden Waste. BWUC: biowaste from university canteen. SA: School of Architecture. PF: Philology Faculty. SF: Science Faculty. ^b^ Heavy metals concentration is in mg/kg.

**Table 3:** Operational characteristics of the second composting batch in the Philology Faculty.

Parameter	Amount
Biowaste from university canteens (BWUC) (kg)	322.5
Garden waste (GrW) (kg)	38.0
Total Input (kg)	360.5
Ratio BWUC/GrW (mass)	8.5:1
Ratio BWUC/GrW (vol.)	1:1
Average Feed (kg BWUC/load)	7.7
Number of Loads	35
Operational days Step 1 (load)	63
Additional Operational Days Thermophilic-mesophilic	60
Max Temperature Reached (°C)	71.8
Time at Temperature > 65 °C (days)	4
Time at Temperature > 55 °C (days)	18
Average Thermophilic Temperature (11–92 d, °C)	51.5 ± 9.5
Average Ambient Temperature (11–92 d, °C)	14.6 ± 3.6
*Rottegrade* Stability, Day 84	Class II
*Rottegrade* Stability, Day 129	Class V

**Table 4:** Physical parameters of samples from dynamic and static composters.

	Initial Degradation Phase (Parallel Experiment)				Steady State Two Stage System	
	Dynamic Composter		Static Composter			
Composter ^a^	DC1_14_	DC1_29_	SC1050_14_	SC1050_29_	DC2_345_	SMC_345_
Day of Operation	14	29	14	29	345	345
Dry Bulk Density, BD_dry_ (g/cm^3^)	0.20 ± 0.00	0.26 ± 0.02	0.18 ± 0.01	0.18 ± 0.01	0.19 ± 0.00	0.20 ± 0.01
Wet Bulk Density, BD_wet_ (g/cm^3^)	0.79 ± 0.00	0.90 ± 0.04	0.57 ± 0.01	0.60 ± 0.03	0.63 ± 0.02	0.74 ± 0.03
Contraction Value, Cv (%)	5.1 ± 2.2	7.9 ± 0.03	6.4 ± 4.0	8.8 ± 2.7	14.3 ± 3.9	12.5 ± 3.9
Particle Density, PD (g/cm^3^)	1.69 ± 0.06	1.71 ± 0.02	1.76 ± 0.08	1.83 ± 0.03	1.68 ± 0.02	1.81 ± 0.09
Total Porosity, ϕ (%)	89.6 ± 0.2	87.0 ± 0.9	91.9 ± 0.3	92.7 ± 0.3	89.9 ± 0.3	91.8 ± 0.5
Air Capacity, AC (%)	30.4 ± 0.5	22.8 ± 3.6	52.3 ± 0.7	50.1 ± 2.3	46.5 ± 1.3	37.4 ± 2.4
Water Holding Capacity, WHC (%)	59.2 ± 0.7	64.3 ± 2.8	39.5 ± 0.4	42.6 ± 2.1	43.4 ± 1.0	54.4 ± 1.9
Free Air Space, FAS (%)	28.0 ± 0.1	28.6 ± 0.3	56.5 ± 0.2	44.7 ± 0.1	32.1 ± 0.1	28.0 ± 0.1
Volatile Solids, VS (%)	80.4 ± 2.6	77.2 ± 0.7	71.2 ± 2.7	63.2 ± 0.7	81.5 ± 1.1	65.0 ± 2.5
Moisture Content (%)	68.7 ± 1.5	67.2 ± 0.4	38.4 ± 0.9	51.8 ± 3.4	64.3 ± 0.3	69.5 ± 0.8

^a^ Samples from DC1 and SC1050 corresponded to the parallel experiment with the same feeding to both composters, as indicated in Section 2.4. Samples from DC2 and SMC corresponded to the steady state operation of the overall composting system at the School of Architecture constituted by the two-stage DC + SMC.

**Table 5:** Coefficients R^2^ for linear regression between variables.

Parameter ^a^	BDdry	BDwet	Cv (%)	PD	ϕ	AC	WHC	FAS	VS	MC
BDdry		0.809 *	−0.013	−0.168	−0.823 * ^c^	−0.772 *	0.695 *	−0.343	0.163	0.303
BDwet	0.809 *		−0.047	−0.123	−0.648 * ^c^	−0.993 **	0.983 **	**−0.580 ^d^**	0.120	**0.550 ^d^**
Cv (%)	0.032	−0.016		0.006	0.016	0.053	−0.058	−0.097	−0.005	0.107
PD	−0.276	−0.057	0.001		0.569	0.017	−0.102	0.146	−0.9996 **	−0.120
ϕ	−0.580	−0.260	−0.001	0.904 * ^b^		0.667 * ^c^	−0.552	0.350	−0.563	−0.303
AC	−0.845 *	−0.992 **	0.021	0.106	0.34		−0.986 **	**0.611 ^d^**	−0.165	**−0.577 ^d^**
WHC	0.765 *	0.997 **	−0.027	−0.04	−0.222	−0.983 **		**−0.617 ^d^**	0.100	**0.591 ^d^**
FAS	−0.886 * ^b^	−0.691	−0.157	0.105	0.351	−0.689	−0.649		−0.152	−0.997 **
VS	0.283	0.059	−0.001	−0.9997 **	−0.909 * ^b^	−0.109	0.041	−0.113		0.126
MC	0.869 * ^b^	0.691	0.161	−0.086	−0.319	−0.684	0.650	−0.999 **	0.093	

^a^ Parameters abbreviations in Table 4. Above the diagonal: Coefficients for linear regression with all data (*n* = 6). Under the diagonal: Coefficients for linear regression excluding the DC1_29_ sample (*n* = 5). **^b^** The significant correlation of DBdry with FAS and with MC for *n* = 5 was lost when DC1_29_ sample was included. The same occurred for the correlation of ϕ with PD and with VS. ^c^ The significant correlation of ϕ with DBdry, DBwet, or AC for all samples was lost when DC1_29_ sample was excluded. ^d^ Underlined: cases of clear nonlinear correlation for the full range of variable values. ** Significant correlation at *p* = 0.01 level. * Significant correlation at *p* = 0.05 level.

**Table 6:** Nutrient elements content, C/N ratio, and pathogen indicators in composts from the UDC.

Compost	Mg (g/kg)	P (g/kg)	Ca (g/kg)	K (g/kg)	TOC (%)	N (%)	C (%)	C/N	R. Class ^a^	SOUR ^b^	Sal. ^c^	FaC. ^d^
SA	2.78 ± 0.25	5.31 ± 1.70	101.0 ± 38.2	11.95 ± 2.19	33.32 ± 4.07	2.49 ± 0.04	37.68 ± 3.56	15.15 ± 1.21	IV	0.95 ± 0.10	0.0 ± 0.0	321 ± nd
PF	3.45 ± 0.35	8.65 ± 3.18	81.7 ± 15.9	19.70 ± 6.51	35.42 ± 2.38	3.58 ± 0.64	38.45 ± 2.76	10.98 ± 2.72	V	0.73 ± 0.06	0.0 ± 0.0	nd

^a^*Rottegrade* Class. ^b^ SOUR: specific oxygen uptake rate (mgO_2_/gVS*h). ^c^ Sal.: salmonella (cfu/gr), ^d^ FaC.: fecal coliform (cfu/gr).
